# Reduced expression of dopamine D2 receptors on astrocytes in R6/1 HD mice and HD post-mortem tissue

**DOI:** 10.1016/j.neulet.2021.136289

**Published:** 2022-01-10

**Authors:** Kate L. Harris, Sarah L. Mason, Benjamin Vallin, Roger A. Barker

**Affiliations:** b Department of Clinical Neurosciences, John van Geest Centre for Brain Repair, E.D. Adrian Building, Forvie Site, Robinson Way, Cambridge CB2 0PY, UK; a MRC Laboratory of Molecular Biology, Cambridge Biomedical Campus, Francis Crick Avenue, Trumpington, Cambridge CB2 0QH, UK; c MRC-WT Cambridge Stem Cell Institute, University of Cambridge, Cambridge, UK

**Keywords:** Neuroscience, Dopamine, Dopamine receptors, Astrocytes, Huntington’s disease, Neurodegeneration, Dopamine D2 receptor

## Abstract

Dysfunction of the central dopaminergic system is thought to contribute to some of the clinical features of Huntington’s disease (HD), and dopamine (DA) receptor antagonists are commonly used to good effect in its treatment. It is well established that there is an early significant reduction in neuronal D2 receptors in HD, considered to be a compensatory response to increased dopaminergic activity. However, no studies have examined the expression of D2 receptors on astrocytes which is important given that these cells have been shown to play a role in the pathogenesis of HD, as well as express dopamine receptors and modulate DA homeostasis in the normal brain. We therefore sought to investigate the expression of D2 receptors on astrocytes in HD, and found them to be reduced in both the R6/1 HD mouse model, and in human post-mortem brain in comparison to controls, suggesting that astrocytes may be important in DA-dependent aspects of HD. Further studies are needed to determine the functional significance of this finding.

## Highlights

• Astrogliosis is seen in the human post mortem hippocampus.
• Astrocytes in human HD hippocampus have reduced D2 receptor expression.
• Astrocytes in R6/1 mouse striatum and hippocampus have reduced D2 receptor expression.

## 1 Introduction

Huntington’s disease (HD) is an autosomal dominant inherited neurodegenerative disorder caused by an expansion of the CAG repeat in the huntingtin gene. Dysfunction of dopamine (DA) signalling is thought to arise early in the disease process and to drive several of the clinical manifestations of HD, including some of the motor and cognitive impairments [1]. In addition, these alterations in dopamine function have been linked to disease pathogenesis as they are associated with free radical production and neuronal death in HD rodent models [2], [3], [4]. Positron emission tomography (PET) imaging studies in HD patients show that there is a progressive reduction in DA receptors in the striatum [5], [6], which begins in premanifest disease stages [5], [7], [8], [9], [10], and is hypothesised to be a compensatory response to increased DA neurotransmission. Furthermore, early studies showed that it was the D2-like subclass of DA receptor (D2R), which typically exerts inhibitory effects on neurons, that were most vulnerable in HD [8], [11], the loss of which correlated with UHDRS motor scores [7], [12] and cognitive performance [13], [14], [12], [15]. Consistent with HD patient findings, HD mouse models exhibit significant reductions in D2R density in the striatum, cortex and dentate gyrus which occurs prior to overt features of disease onset [16], [17], [18], [19].

While the impact of DA on neuronal signalling has been relatively well studied, emerging evidence suggests that astrocytes may also play an important role in DA homeostasis. Astrocytes express both D1-like and D2-like DA receptors [20], [21], [22], and enzymes involved in the transport and degradation of dopamine, such as VMAT2 and MAO [23], [24]. Furthermore, it has been shown that the selective deletion of DA receptors on astrocytes leads to increased neuroinflammation and altered glutamate signalling [25], [26], [27], whilst the application of DA to isolated astrocyte cultures elicits the release of neurotrophins GDNF and NGF [28]. In addition, DA and DA agents provoke increased intracellular calcium transients in astrocytes [29] which have recently been shown to induce synaptic depression in the nucleus accumbens in mice [30].

This is especially relevant to HD, as astrocytes have now been shown to be affected by the disease process [31], [32], [33], [34] and to possibly be involved in disease pathogenesis [35], [36]. Furthermore, HD astrocytes exhibit reduced levels of the Kir4.1 potassium ion channel [37] as well as the glutamate transporter GLT1 [38], leading to impairments in potassium and glutamate uptake. It is therefore possible that HD astrocytes also display alterations in regard to dopamine, and thus we sought to address this using a transgenic mouse model of HD as well as human HD post-mortem tissue. We focussed on the striatum and hippocampus given their central role in HD [39] and found that the expression of D2-like receptors is reduced in HD mice and HD post-mortem tissue at both sites compared to controls.

## 2 Methods

### 2.1 Animals

R6/1 mice were purchased from the Jackson Laboratory (USA) and the colony was maintained by backcrossing to B6CBAF1/J females purchased from Harlan Ltd (UK). Genotyping was performed by Laragen Inc. (USA). The mice were kept in standard cages on a twelve-hour light–dark cycle and were used for experiments at 6 months of age. Food and water was available *ad libitum*. All experiments were performed in compliance with the regulations of the UK Home Office and in accordance with the UK Animals (Scientific Procedures) Act of 1986.

### 2.2 Post- mortem human study in HD and control tissue

The study received ethical approval from London-Bloomsbury Research Ethics Committee (REC reference 16/LO/0508). The brain tissue was obtained from the Cambridge Brain Bank (Cambridge, UK). Tissue preparation was performed by the Brain Bank, during which the brains were formalin-fixed and embedded in paraffin in 10um-thick coronal sections. The brain tissue contained the anterior hippocampus from adult-onset HD patients (n = 11) and aged- matched controls (n = 10).

### 2.3 Immunofluorescence staining of mouse tissue

Mice were sacrificed by cervical dislocation and the brains immediately removed, post-fixed overnight in 4% paraformaldehyde (PFA) and then cryoprotected in phosphate buffer saline (PBS) containing 30% sucrose for 2 days. The brains were then cut into 40 μm free floating coronal sections (one in 12 series) using a vibratome and transferred to a 24-well plate for immunostaining. Following 3 washes with 0.2% Triton X-100 in PBS (PBST), the sections were incubated in blocking solution containing 5% goat serum in PBST for one hour and then incubated with the primary antibodies (GFAP Abcam ab7260 1:1000, S100B Sigma-Aldrich S2352 1:500, D2R Santa Cruz SC5303 1:200, D2R Merck Millipore AB5084P), in blocking serum overnight at 4 °C. After three five-minute washes in PBST, the sections were incubated for 2 h with the appropriate secondary antibodies at room temperature in the dark. Following three more washes, sections were incubated for 10 min with DAPI (1:1000, Sigma-Aldrich). Finally, sections were washed a further 3 times in PBST and then mounted using vectashield onto 1% gelatin- coated slides for imaging. As a negative control, some sections were incubated without the primary antibody.

### 2.4 Immunofluorescence staining of post-mortem tissue

Sections were oven- dried at 60 °C and left overnight in 100% xylene in order to remove the paraffin (Fisher Chemical X/ 0250, 17). The next day, sections were transferred to 100% EtOH, 90% EtOH, 70% EtOH and dH_2_0 for 10 min each to rehydrate the tissue. Antigen retrieval was then performed in 10 mM sodium citrate buffer (Sigma Aldrich S4641), pH = 6 for 20 min. Next, sections were washed in PBST for 5 min. Sections were then stained using the procedure described above. Prior to coverslipping, sections were incubated in Sudan black (Abcam, ab146284) for 15 min followed by 3 washes in PBST in order to reduce autofluorescence [40].

### 2.5 Immunohistochemistry staining of post-mortem tissue

Sections were prepared in the same way as for the human immunofluorescence staining and were then incubated overnight in the primary antibody for GFAP (1:1000, abcam, ab7260). The following day, sections were washed three times in PBST before being incubated with a biotinylated secondary antibody for 2 h. Sections were then incubated in ABC solution (Vector Laboratories PK-6100) for 1 h at room temperature, before being rinsed 3 times with PBST. Di-aminobenzidine (DAB) solution (Vector Laboratories SK-4100) was used for immunohistochemical development and was applied to the tissue for approximately 30 s. The sections were then rinsed with dH_2_0, and counterstained with filtered Hematoxylin (Thermo Scientific 6765004) for 30 s. Excess hematoxylin was removed by dipping the sections in 1% acid alcohol. Finally, sections were dehydrated in dH_2_0, 70% EtOH, 90% EtOH and 100% EtOH for 10 min each. Sections were then left in 100% xylene overnight, before being mounted with DPX mounting medium (Thermo Scientific LAMB/ DPX).

### 2.6 Microscopy

Images of GFAP and D2R immunofluorescence were captured with a confocal Leica SPE microscope. For the mouse tissue, five z-stack images (total thickness 15 μm) were taken per region of interest (CA1, CA3 and DG region of the hippocampus and the striatum) at 40x magnification. For the human post-mortem tissue, 10 z-stack images (total thickness 5 μm) were acquired at 40x magnification in the CA1 region of the hippocampus. Post-mortem tissue sections which were stained for GFAP using immunohistochemistry were sent to the Histopathology/HIS facility at the Cancer Research UK Cambridge Institute and were imaged using an Aperio Scanscope AT2 (Leica Biosystems) at a 20x magnification, with a resolution of 0.503 µm per pixel.

### 2.7 Quantification

#### 2.7.1 Quantification of the number of D2R+ astrocytes per image was performed with ImageJ

To do so, the multi point tool was used to manually count the absolute number of cells positive for DAPI, GFAP and D2R per mm^2^ (GFAP, D2R & DAPI positive cells/mm^2^), and the average was calculated for each brain. The percentage of astrocytes positive for D2R was also calculated (GFAP, D2R & DAPI double positive cells / DAPI cells) * 100]. Total D2R staining was also performed in a similar manner (D2R & DAPI positive cells / mm2) and (D2R & DAPI double positive cells / DAPI cells) * 100]. Aperio ImageScope software was used to quantify the brown (DAB) immunohistochemical staining of GFAP. In order to do this, the annotation tool was used to manually outline the CA1 region of the hippocampus, including only the grey matter. Next, the Positive Pixel Count V9 algorithm was selected in order to quantify the DAB staining. This algorithm results in segmentation of each pixel in the ROI with it being classed as either weakly positive (yellow), medium positive (orange) strongly positive (red) or background (blue) [as described in 41]. To account for differences in the size of region of interest between cases, the staining is reported as the total positive pixels (medium and strong) per mm^2^ to give the positive pixel count/mm^2^. Quantification was performed in a blinded manner.

### 2.8 Statistical analysis

The data followed a normal distribution and therefore unpaired t-tests were used to compare immunostaining in WT and R6/1 mice and the HD and control post-mortem tissue. For the post-mortem tissue, a simple linear regression was also used to investigate whether there was a correlation between D2R expression and disease stage or age. Graphpad prism software V9 was used for data analysis. Data are expressed as mean ± standard deviation. Values of P < 0.05 were considered as statistically significant.

## 3 Results

**Fig. 1:**
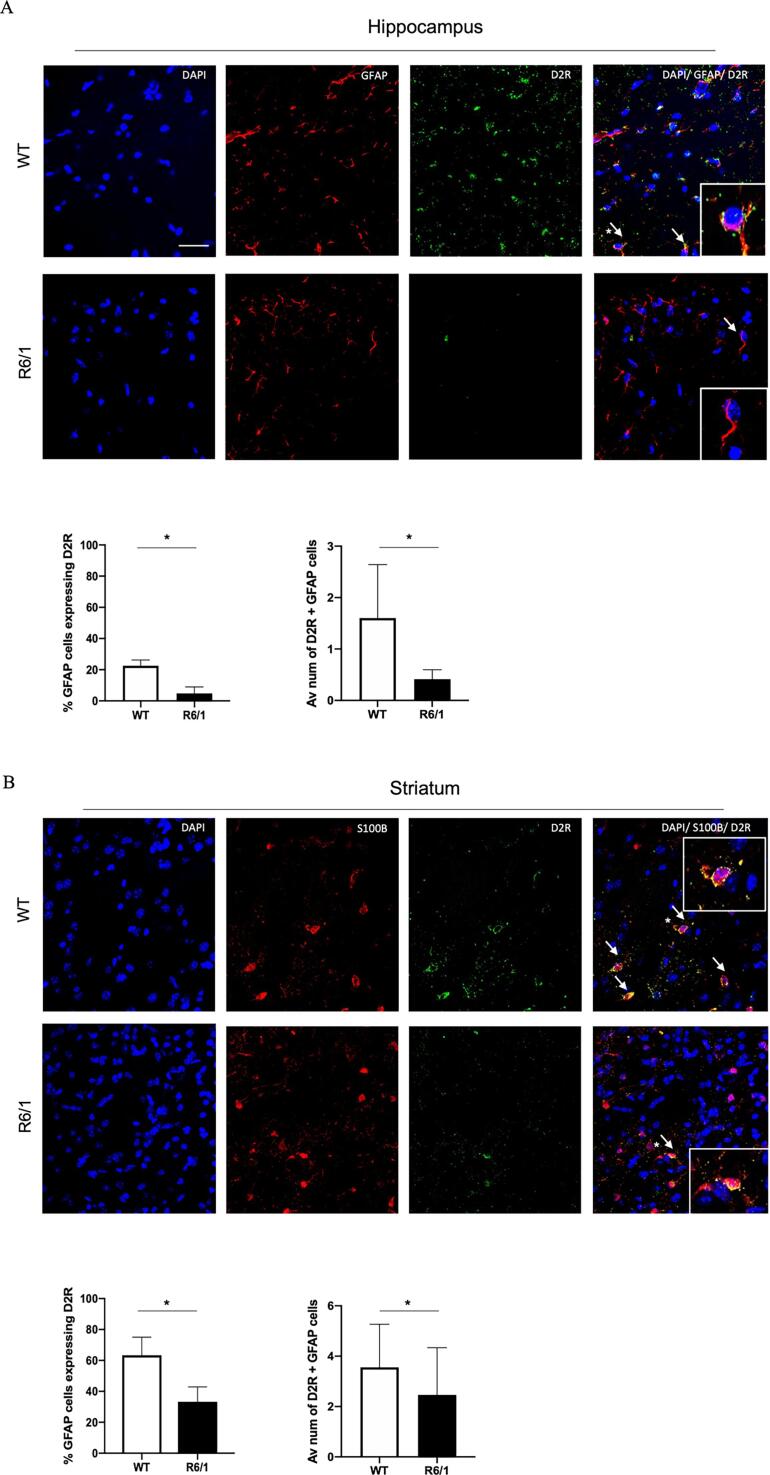
Quantification of D2R+ astrocytes per mm^2^ in the hippocampus and striatum of R6/1 and WT mice. R6/1 mice exhibit fewer D2+ astrocytes compared to WT mice. (A) Quantification of the GFAP+ astrocytes expressing D2R in the hippocampus and (B) S100B+ astrocytes expressing D2R in the striatum. Plots represent % of GFAP+ cells expressing D2R and number of cells positive for DAPI, GFAP and D2R per mm2. Significantly fewer astrocytes expressing D2R were observed in the R6/1 mice compared to WT. N = 4 per group. Shows mean with standard deviation. Arrows indicate D2R+ GFAP cell. Asterix indicates cell shown in the enlarged image. *p < 0.05. Scale bar = 25 μm.

We first investigated whether the astrocytic expression of D2R was reduced in R6/1 mice in comparison with WT littermates by performing double immunostaining for GFAP and D2R. We initially looked at the hippocampus, given we have recently shown that cognitive impairments linked to this structure occur early in patients with HD [42], [43] and that it is a site of early pathology in transgenic HD mice [44]. We found that the astrocytic D2-like receptor expression in the CA1 region of the hippocampus was significantly reduced in manifest R6/1 mice (t(6) = 4.585, p = 0.003, see Fig. 1a). Expression was also reduced in the CA3 and DG hippocampal regions (data not shown). We also wished to confirm that this was not just an abnormality found at this brain site by also looking at the striatum in these mice. For this we used s100b to label astrocytes instead of GFAP because the gene encoding glial fibrillary acid protein (GFAP) is not highly expressed in striatal astrocytes [43]. The results confirmed that D2-like receptors were less abundant on astrocytes in the striatum of R6/1 HD mice compared with WT controls (t(6) = 2.692, p = 0.036 see Fig. 1b). Interestingly, there was significantly greater baseline astrocytic D2R expression in the striatum compared to the hippocampus for both WT (t(6) = 4.619, p = 0.036) and R6/1 mice (t(6) = 4.380, p = 0.0047), which suggests that astrocytes in the striatum have a greater role in DA homeostasis presumably due to higher ambient DA levels in this region [45]. However, this comparison should be interpreted cautiously as different antibodies were used to label the astrocytes in the hippocampus and striatum.

**Fig. 2:**
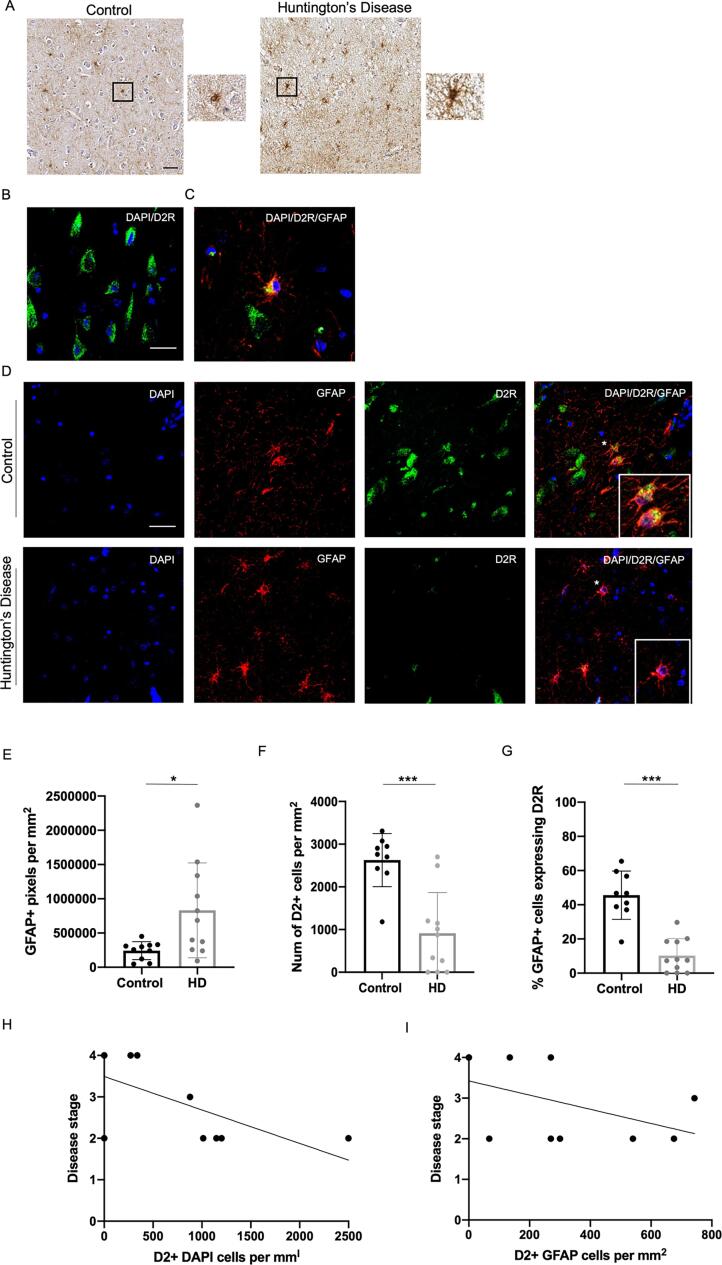
Quantification of DR2R+ GFAP cells in CA1 hippocampal tissue from HD patients and matched- controls. There were significantly fewer D2R+ astrocytes in HD post-mortem tissue compared to controls. (A) Representative images of GFAP+ astrocytes in CA1 region of the hippocampus of a control (left) and a HD brain (right). (B) Control tissue showing D2R and DAPI (C) Control tissue showing D2R, GFAP and DAPI (C). (D) Representative images of GFAP+ astrocytes expressing D2R in the CA1 region of the hippocampus of a control (top) and a HD brain (bottom). (E) Number of GFAP+ cells per mm^2^. (F) Quantification of the total number of D2R+ cells. (G) Percentage of GFAP-positive cells expressing D2R (H) Correlation between total number of D2R-expressing cells and disease stage. (I) Correlation between the proportion of GFAP+ astrocytes expressing D2R and disease stage. Asterix indicates cell shown in the enlarged image. Control n = 9 per group (n = 10 for the immunohistochemical staining), HD n = 11 per group. Shows mean with standard deviation. *p < 0.05. ****p < 0.001. Scale bar = 25 μm for A and 100 μm for B-D.

Based on these findings, we next sought to investigate whether astrocytes exhibit a similar reduction in D2-like receptor expression in human CA1 hippocampal HD post-mortem tissue (see Table 1 for demographic details on the tissue used). We first examined the morphology of astrocytes using an immunohistochemical staining for GFAP and found that HD cases displayed a significant increase in GFAP+ expression (t(19) = 2.639, p = 0.016), alongside an increase in GFAP+ cell area (t(19) = 3.443, p < 0.024), indicative of astrogliosis (see Fig. 2a). We next confirmed that D2R could be visualised in the human hippocampal post-mortem tissue using immunofluorescence labelling (Fig. 2b). A double immunostaining for GFAP and D2R then revealed that some hippocampal GFAP+ astrocytes expressed D2R, as has been previously shown in the human prefrontal cortex [31], [46], with such cells often being located in close proximity to a D2R+ neuronal cell (see Fig. 2b). Importantly, HD cases displayed a significant reduction in D2-like receptor expression compared to controls both in terms of total expression (i.e., including all Dapi+ cells which expressed D2R) (t(18) = 4.631, p < 0.001, see Fig. 2d) and on GFAP+ astrocytes specifically (t(18) = 6.549, p < 0.001, Fig. 2e). For HD cases, there was a significant negative correlation between the total number of cells expressing D2R and disease stage (r = 0.641, *p* = 0.046, Fig. 2f), with a similar trend for the number of GFAP+ astrocytes expressing D2R and HD stage (r = 0.474, *p* = 0.166, Fig. 2g). However, there was no correlation between total GFAP expression and astrocytic D2R expression (r = 0.129, *p* = 0.587). Furthermore, there was no correlation between age and astrocytic D2R expression for either HD patients (r = −0.273, *p* = 0.446) or controls (r = −0.480, *p* = 0.191).

**Table 1:** Control and HD case demographics.

Patient ID	Control (n = 9)	HD (n = 11)
Age at death	62.1 (6.9)	62.9 (13.5)
% male	50%	54.5%
Vonsattel disease stage	N/A	3 (0.9) n = 5 stage 2, n = 1 stage 3, n = 4 stage 4

## 4 Discussion

It has been known for many years that there is a loss of striatal DA receptors prior to a clinical diagnosis of HD in patients, which then contributes to both motor and cognitive impairments. While these changes have been thought to occur exclusively in neurons, recent research has shown that astrocytes also play an important role in DA homeostasis as well as HD pathogenesis. Therefore, the current study assessed the expression of DA receptors in astrocytes in a well validated murine model of HD and in HD patient tissue. We found that there is a significant reduction in the expression of D2-like receptors on astrocytes in the hippocampus and striatum in HD mice which was confirmed in HD human hippocampal post-mortem tissue.

The cause of this reduction in glial D2R in HD remains unclear. The loss of D2R on neurons in HD has been hypothesized to be a compensatory response to increased dopamine signaling which occurs in the early stages of the disease [47], and this may also be the case for astrocytes. HD astrocytes may also have become reactive and lost their supportive function, which is known to alter expression of key astrocyte proteins [48]. Indeed, we found evidence for astrogliosis in the HD hippocampal post-mortem tissue, which is consistent with previous findings [49], [50], [37]. However, we did not observe a correlation between GFAP+ astrocyte expression and the extent of D2 loss, although other markers of astrogliosis such as C3 and GBP2 were not looked into nor whether the downregulation of D2R in HD starts in neurons or glia or both at the same time.

Our studies concentrated on the hippocampus given previous work showing it is affected in HD patients as well as transgenic mice. In rodents, it is known that the pharmacological or genetic inhibition of hippocampal D2R inhibits LTP maintenance [51] impairs spatial memory performance [52], [53], [54], and reduces the firing rate and spatial tuning of hippocampal place cells [55], [56]. Thus, the reduction in hippocampal D2R observed in the current study may contribute to reported disturbances in hippocampal synaptic plasticity in HD mice [44], as well as to the spatial memory deficits observed in HD patients [42], [43]. It is possible that the specific loss of D2R from astrocytes also contributes to synaptic dysfunction in HD, as a recent study has shown that the DA- induced activation of D1R on astrocytes in the nucleus accumbens depresses excitatory synaptic transmission [30]. However, similar effects have not yet been demonstrated for D2-like receptors or in other brain regions of relevance to HD, such as the dorsal striatum, cortex or hippocampus. Nevertheless, D2 antagonists reduce calcium transients in astrocytes [29] suggesting that the activation of these receptors may indeed influence synaptic transmission.

Whilst a direct role for astrocytic D2R in synaptic plasticity has not yet been established, there is evidence to suggest they are involved in the regulation of neuroinflammation. For example, the selective deletion of astrocytic D2R in mice leads to increased expression of activated microglia and pro-inflammatory cytokines, a downregulation of the anti-inflammatory small heat-shock protein αB-crystallin, and increased dopaminergic neuronal loss following stress [27]. The pharmacological inhibition of D2R also leads to elevated Ca^2+^ transients in ventral midbrain astrocytes in response to glutamate application [25], suggesting that astrocytic D2Rs may attenuate glutamatergic transmission in a similar manner to neuronal D2Rs [57], [58]. It is therefore likely that the loss of astrocytic D2R contributes to neuroinflammation and Ca^2+^ induced excitability and neurotoxicity in HD, which may explain why manipulating the glial compartment impacts on the expression and development of HD in rodents [35]. The loss of astrocytic D2R in neurodegeneration, as highlighted by the current study has clinical implications for other neurological conditions which are characterised by alterations in dopamine transmission such as Parkinson’s disease (PD) and schizophrenia.

There are a number of limitations to the present study first, and foremost the fact that we couldn’t investigate the functional significance of the loss of these astrocytic D2 receptors in HD. Furthermore, the sample size of the post-mortem cases was relatively small, and we did not have access to striatal post-mortem tissue to replicate our findings from the HD mice. Additionally, it is not known whether the HD patients in the post-mortem study were taking dopamine blocking agents, which could have influenced the expression levels of astrocytic D2R. However, studies in rodents suggest that antipsychotic treatment is in fact associated with an upregulation of D2R, either due to an increase in synthesis of D2R or a decrease in the degradation of the receptor [59], so if this were the case, we may have expected to observe an increase in astrocytic D2R in HD. Seeing D2 antagonists are commonly used in HD, future studies should investigate the impact of this medication on DA signalling in astrocytes in HD.

## 5 Conclusion

This study is the first to explore dopamine receptor expression in astrocytes in animal models of HD and patient post-mortem material and we found that there is a clear downregulation of D2R on astrocytes in both cases. The functional significance of this awaits clarification, but does offer a potential new disease mechanism which might contribute to dopaminergic dysfunction in HD and warrants further investigation, especially given that dopamine acting drugs are currently widely used to treat patients with HD in the clinic.

## CRediT authorship contribution statement

**Kate L. Harris:** Methodology, Writing – original draft. **Sarah L. Mason:** Writing – review & editing. **Benjamin Vallin:** Writing – review & editing. **Roger A. Barker:** Writing – review & editing.
